# Acute Post-traumatic Invasive Basidiobolomycosis With Concomitant Mucormycosis in the Southeastern United States

**DOI:** 10.7759/cureus.116281

**Published:** 2026-09-15

**Authors:** Harrison Dai, Lili Tao

**Affiliations:** 1 Department of Pathology, Microbiology, and Immunology, Vanderbilt Health, Nashville, USA

**Keywords:** basidiobolomycosis, basidiobolus, entomophthorales, invasive fungal disease, post-traumatic fungal infection

## Abstract

Basidiobolus spp., belonging to the order Entomophthorales, cause a rare group of fungal infections known as basidiobolomycosis, which has been described as causing subcutaneous and gastrointestinal clinical manifestations in both immunocompetent and immunocompromised patients. Basidiobolomycosis is thought to be endemic to subtropical and tropical regions such as Asia, Africa, South America, and the southwestern United States. Herein, we describe a case of tissue-invasive Basidiobolus infection with concomitant mucormycosis in a middle-aged woman following a traumatic motor vehicle accident in the southeastern United States.

## Introduction

Basidiobolomycosis is a rare, emerging fungal infection caused by Basidiobolus spp. that can affect multiple organ systems, from the subcutaneous tissue to the deep gastrointestinal tract. Basidiobolomycosis infections have been reported mainly affecting tropical climates within several continents, including Asia, Africa, and South America [1,2]. In North America, isolated cases of Basidiobolomycosis have been described in the southwestern States, such as Arizona. Clinically, basidiobolomycosis typically presents as an indolent, chronic, and slowly progressing infectious process. Infections within humans are generally believed to occur following traumatic inoculation. While invasive fungal infections such as mucormycosis are recognized potential complications following severe trauma, acute pathogenic basidiobolomycosis infections have rarely been described, let alone reported in the southeastern United States. Herein, we describe an unusual coinfection of tissue invasive basidiobolomycosis and mucormycosis in a middle-aged woman following a traumatic amputation secondary to a motor vehicle accident within Middle Tennessee.

## Case presentation

A 56-year-old female patient with unclear past medical history presented to the emergency department following a motor vehicle accident that resulted in a near-complete traumatic amputation of her right lower extremity, accompanied by polytrauma involving multiple bodily sites. Emergency surgical interventions included right lower extremity amputation and exploratory laparotomy for intra-abdominal injuries. Despite extensive debridement, on day 5 of admission, the patient developed necrotic tissue at the amputation site, a fever of 39.6 °C, and septic shock. Her white blood cell (WBC) count was markedly elevated at 24.3 x 10^9^/L (normal range: 3.9-10.7 x 10^9^/L) with neutrophil predominance. Empirical antimicrobial therapy was initiated with intravenous vancomycin (15 mg/kg every eight hours) and cefepime (2 g every eight hours).

Initial bacterial cultures from the necrotic tissue and blood collected on day 6 grew *Stenotrophomonas maltophilia*. The antimicrobial regimen was adjusted to include vancomycin (20 mg/kg/day), ceftazidime/avibactam (2.5 g every eight hours), and aztreonam (2 g every eight hours). Subsequently, the antimicrobial regimen was modified to vancomycin, levofloxacin (750 mg daily), and metronidazole (500 mg every eight hours) based on susceptibility results. Despite intensive coverage for bacterial infection, the patient remained febrile; the amputation site appeared to be necrotic and produced a large amount of bloody/brown discharge from the amputation site. However, her WBC count gradually improved and returned to the normal range on day 10.

On day 12, the patient underwent further surgical debridement due to extensive necrosis of the skin flap and muscle tissue. The necrotic tissue was sent for bacterial and fungal culture, along with histopathology examination. Histopathology of the amputation site revealed extensive necrosis, acute inflammation, and the presence of broad, pauciseptate ribbon-like hyphae with tissue invasion, along with zygospores at the edge of the tissue (Figures 1A, 1B). The specimen was inoculated on Sabouraud dextrose agar (SDA), mycobiotic agar, and brain heart infusion agar supplemented with gentamicin and cycloheximide and incubated at 30°C for fungal culture according to routine procedure. After two days of incubation, two distinct mold colony types developed on the SDA plate and were subcultured for further identification. One mold formed cream-colored, waxy colonies with a cerebriform center and radial fissures in the outer zone (Figure 1C). Lactophenol blue staining demonstrated broad, pauciseptate hyaline hyphae with round, smooth, thick-celled zygospores exhibiting beak-like appendages (Figure 1D). These morphologic characteristics are consistent with Basidiobolus spp. The other mold recovered from the same culture was identified as Mucor spp. based on macroscopic and microscopic phenotypic features. A separate deep clot fungal culture collected on day 13 again isolated Basidiobolus spp. The patient was therefore diagnosed with invasive basidiobolomycosis and mucormycosis. Antifungal therapy was then initiated with liposomal amphotericin B (5 mg/kg/day). Unfortunately, due to multifactorial shock and organ failure, the patient expired on day 17 as a result of her condition.

**Figure 1:**
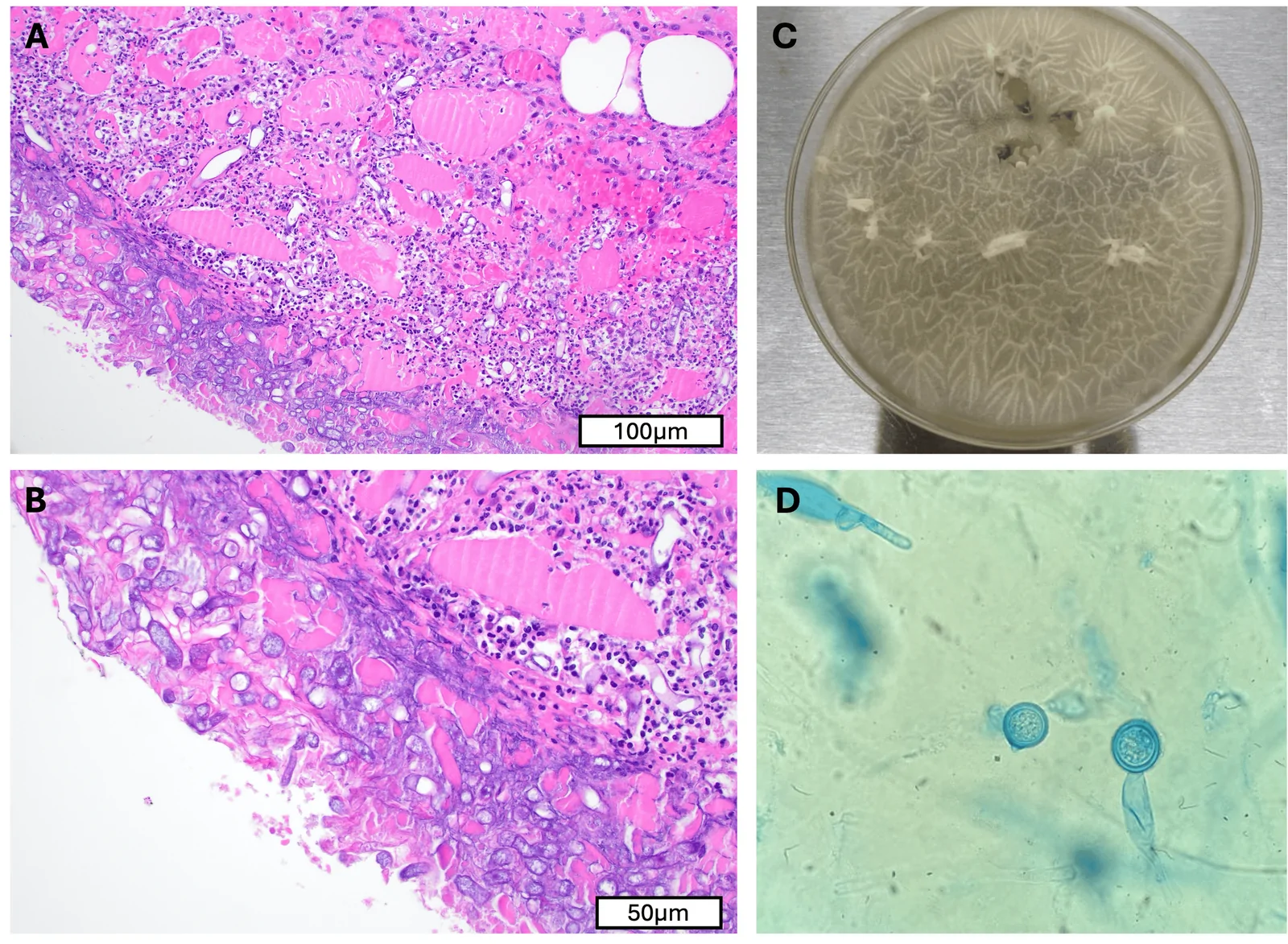
Histopathology and microbiology examination of Basidiobolomycosis (A) Histologic sections demonstrating broad, pauciseptate hyphae and zygospores in deep soft tissue. Hematoxylin and Eosin, 100× magnification. (B) Broad, pauciseptate hyphae and zygospores present within deep soft tissue. Hematoxylin and Eosin, 400× magnification. (C) A fungal culture demonstrating cream-colored, waxy colonies with a cerebriform center and radial fissures in the outer zone. (D) Lactophenol blue staining demonstrating broad, hyaline hyphae with round, smooth, thick-celled zygospores exhibiting beak-like appendages

## Discussion

Basidiobolus spp. are saprophytic fungi that belong to the order Entomophthorales and are responsible for a rare group of fungal infections known as basidiobolomycosis. These fungi are commonly found in soil, decaying vegetation, and the intestinal tract of amphibians and reptiles, as well as in their excreta [1-3]. Infection with Basidiobolus spp. is believed to occur following traumatic inoculation of fungal spores into the skin or mucosal surfaces [3,4]. Clinically, basidiobolomycosis has most commonly been described as presenting with cutaneous and subcutaneous tumor-like growths that develop slowly and may be ulcerated or hyperpigmented [1,3]. However, clusters of cases with gastrointestinal manifestations or renal involvement have also been reported in the literature [1,5-7]. Although the infection is typically indolent and slowly progressive, basidiobolomycosis can pose significant diagnostic challenges because of its rarity and nonspecific clinical presentation.

Diagnosis of basidiobolomycosis relies on histopathologic examination and fungal culture of infected tissue [1,3-5,7]. Histology typically reveals extensive fibrosis and an inflammatory response containing eosinophils in chronic cases. A reddish blanket of eosinophilic material may surround short, ribbon-like hyphae, a phenomenon known as the Splendore-Hoeppli phenomenon. Fungal cultures typically yield rapidly growing, cream-colored, waxy colonies with distinctive radial fissures. Microscopic examination shows vegetative hyphae and characteristic “beaked” zygospores, which are diagnostic for Basidiobolus spp. *Basidiobolus ranarum* is responsible for most cases of human basidiobolomycosis, although infections caused by other species have also been reported.

In our case, the patient presented with acute tissue necrosis following a vehicle accident, which is not typically seen with basidiobolomycosis. Histopathologic examination showed a neutrophilic inflammatory response more consistent with an acute infectious process, rather than the fibrotic and eosinophil-rich tissue response commonly observed in chronic infection. Although hyphal morphology on histopathology can be similar between Basidiobolus spp. and Mucor spp., and Mucor sporangia can mimic zygospores, sporangia are uncommonly seen on histopathology, particularly in deep tissue specimens. Therefore, the presence of zygospores in deep tissue supported invasive Basidiobolus infection.

Fungal cultures from specimens collected in the operating room at multiple time points repeatedly isolated Basidiobolus despite extensive debridement. Given the classic macroscopic and microscopic features observed in culture, further identification by matrix-assisted laser desorption/ionization time-of-flight mass spectrometry was not pursued, and species-level identification by sequencing was not attempted.

While post-traumatic mucormycosis has been reported and usually requires extensive debridement or amputation, acute basidiobolomycosis after a vehicle accident has not been documented to the best of our knowledge [8-11]. It is possible that the extensive tissue damage due to the vehicle accident, along with concurrent bacterial and Mucor infections, facilitated tissue invasion by Basidiobolus. Because of the concurrent Mucor and bacterial infection, it is difficult to determine whether Basidiobolus played a central role in the extensive soft-tissue infection. However, Basidiobolus spp. was repeatedly isolated from different specimens after extensive debridement, suggesting that the fungus persisted in the tissue and may have contributed to the infection. Because the patient sustained severe multisystem traumatic injuries, the extent to which infection contributed to death is unclear; however, it clearly complicated the subsequent clinical course and management.

Although Basidiobolus spp. are commonly found in soil, Basidiobolomycosis is considered endemic to subtropical and tropical regions, including Asia, Africa, South America, and the southwestern United States [2]. In the United States, clusters of cases have been predominantly reported in southwestern states such as Arizona and Utah [6,12]. However, cases of basidiobolomycosis have not yet been reported in the Southeastern United States at the time of this report. Therefore, Basidiobolus was not considered a potential cause of infection in this case until it was repeatedly isolated from fungal culture after extensive debridement, underscoring the importance of conventional culture in identifying the causative organism.

## Conclusions

Basidiobolus spp. may lead to soft-tissue infection after vehicle accidents probably by traumatic inoculation beyond traditional geographic regions. Histopathology and fungal cultures are essential in diagnosing basidiobolomycosis.
